# The role of SRGN in the survival and immune infiltrates of skin cutaneous melanoma (SKCM) and SKCM-metastasis patients

**DOI:** 10.1186/s12885-020-06849-7

**Published:** 2020-05-05

**Authors:** Xiaofang Wang, Hui Xiong, Daning Liang, Zhenzhen Chen, Xiqing Li, Kun Zhang

**Affiliations:** 1 Department of Dermatology and Venerology, University of Chinese Academy of Sciences-Shenzhen Hospital, Shenzhen, China; 2 Department of Dermatology, Sun Yat-sen Memorial Hospital, Sun Yat-sen University, Guangzhou, China; 3 Department of Dermatology, Peking University Shenzhen Hospital, Shenzhen, China; 4 Guangdong Provincial Key Laboratory of Malignant Tumor Epigenetics and Gene Regulation, Sun Yat-sen Memorial Hospital, Sun Yat-sen University, Guangzhou, China

**Keywords:** SRGN, Immune infiltrates, Survival, SKCM, SKCM-metastasis

## Abstract

**Background:**

Skin cutaneous melanoma (SKCM) is one of most aggressive type of cancers worldwide. Serglycin (SRGN) is an intracellular proteoglycan that playing an important role in various tumors. However, its effect on immune infiltrates and whether it associates with survival of SKCM and SKCM-metastasis patients has not been explored.

**Methods:**

We evaluated SRGN expression via the databases of Oncomine, Tumor Immune Estimation Resource (TIMER) and Gene Expression Profiling Interactive Analysis (GEPIA). The influence of SRGN expression on survival of SKCM and SKCM-metastasis patients was analyzed using TIMER database. Furthermore, the correlations between SRGN expression and immune infiltrates or gene marker sets of immune infiltrates were also analyzed via TIMER database.

**Results:**

We found that the expression of SRGN in SKCM and SKCM-metastasis tissues was significantly increased compared to the normal skin tissues (*P* < 0.001). Interestingly, it was showed that lower level of SRGN expression and lower immune infiltrates of B cell, CD8+ T cell, Neutrophil, and Dendritic cell were correlated with poor survival rate of SKCM and SKCM-metastasis patients (*P* < 0.001) but not SKCM primary patients. We also demonstrated that SRGN expression was positively associated with the immune infiltrates and diverse immune marker sets in SKCM and SKCM-metastasis.

**Conclusions:**

Our findings indicated that SRGN was associated with the survival of SKCM and SKCM-metastasis patients. SRGN may be a new immune therapy target for treating SKCM and SKCM-metastasis.

## Background

Skin cutaneous melanoma (SKCM) is a major public health problem worldwide. Despite public health campaigns, the mortality of SKCM patients is still increasing in many countries [1]. To make things worse, there are no effective systemic therapies to treat advanced stages of SKCM [2]. Although the five-year relative survival rate for early stage melanoma is more than 95% [3], the reported survival for stage IV melanoma is rarely longer than a year [2]. Thus, early detection is important and finding out an early prognostic maker will be helpful for improving the survival of SKCM patients.

Recently, numerous studies have shown that the dysfunction of immune system plays a key role in the progression of SKCM [4, 5]. It has been demonstrated that tumor-infiltrating lymphocyte grade is an independent predictor of sentinel lymph node status and survival in SKCM patients [6]. Sara R. Selitsky et al. demonstrated that B cells have important predictive role in SKCM [5]. Therefore, immunotherapy is popular used for treating melanoma. However, even immune checkpoint inhibitors (ICIs) such as antibodies targeting either the cytotoxic T-lymphocyte-associated protein 4 (CTLA-4) or the programmed death 1 (PD1) immune checkpoints, yet approximately 50% of the patients do not respond to treatment [7, 8]. Thus, it is still necessary to find novel immune-related therapeutic targets in SKCM.

Serglycin (SRGN) is a major proteoglycans expressed in hematopoietic cells, endothelial cells, and macrophages [9–11]. It contains a peptide core of 17.6 kDa which is rich in serine-glycine repeats and has eight glycosaminoglycan (GAG) side chains [11]. A series of evidences have demonstrated that SRGN involves in the progression of tumors including acute myeloid leukemia [12], breast cancer [13, 14], colorectal cancer [15], nasopharyngeal carcinoma [16, 17], multiple myeloma [18], and etc. [19]. However, in SKCM, the role of SRGN has not been explored. Whether SRGN playing similar roles in SKCM and the metastatic SKCM that is not known.

In the present study, we firstly comprehensively analyzed the SRGN expression and correlation with the survival of SKCM and SKCM-metastasis patients. Moreover, we investigated the relationship between SRGN expression and the tumor-infiltrating immune cells in SKCM and SKCM-metastasis. Our findings will shed light on the important role of SRGN in SKCM and SKCM-metastasis.

## Methods

### SRGN expression analysis

The expression of SRGN in different tumors and normal tissues were identified in Oncomine database (https://www.oncomine.org/resource/login.html) [20]. It includes 34,219 samples. The threshold was defined as: fold change of 2, *P*-value of 0.0001 and gene rank of top 10%. We also used the Tumor IMmune Estimation Resource (TIMER) database (https://cistrome.shinyapps.io/timer/) to study the differential expression between tumor and adjacent normal tissues for SRGN across all The Cancer Genome Atlas (TCGA) tumors [21]. For comparison of the SRGN expression in SKCM and normal tissues, the Gene Expression Profiling Interactive Analysis (GEPIA) database (http://gepia.cancer-pku.cn/index.html) was used [22]. GEPIA is a web server for analyzing the RNA sequencing expression data of 9736 tumors and 8587 normal samples from the TCGA and the GTEx projects, using a standard processing pipeline.

### Survival analysis

We investigated the association between SRGN expression and survival rate of patients with different tumors using the TIMER database. In this database we also explored the correlation between different tumor immune subsets (B cells, CD4^+^ T cells, CD8^+^ T cells, Neutrphils, Macrophages and Dendritic cells) and survival rate of SKCM and SKCM-metastasis patients.

### Immune infiltrates analysis in SKCM and SKCM-metastasis

TIMER is a comprehensive resource for systematical analysis of immune infiltrates across diverse cancer types [21]. It includes 10,897 samples across 32 cancer types from TCGA to estimate the abundances of six immune infiltrates (B cells, CD4+ T cells, CD8+ T cells, Neutrphils, Macrophages and Dendritic cells). So using the TIMER database, we analyzed the immune infiltrates in SKCM and SKCM-metastasis via SRGN modules with adjustment of clinical factors (age, gender, ethnicity, tumor stages and tumor purity). Tumor purity was inferred from copy number alteration data using an R package and CHAT [23]. Furthermore, we also explored the correlations between SRGN expression and gene markers of tumor-infiltrating immune cells through correlation modules in TIMER database. The gene markers are reported in previous studies [24, 25].

### Statistical analysis

The results generated from Oncomine database were displayed with fold changes, ranks, and *P* values. The SRGN expression of SKCM was also analyzed using GEPIA database. In GEPIA, the |Log2FC| cutoff value is 1.5 while the *P*-value cutoff is 0.001. The Kaplan-Meier plots were drawn with TIMER for immune infiltrates and SRGN to visualize the survival differences. Levels are divided into low and high levels by the split percentage of patients 50%. *P* value of log-rank test for comparing survival curves of two groups is showed in each plot. The correlation of SRGN expression was evaluated by Spearman’s correlation and statistical significance. Values of *P* < 0.05 were considered statistically significant.

## Results

### The SRGN expression in different types of human tumors

**Fig. 1:**
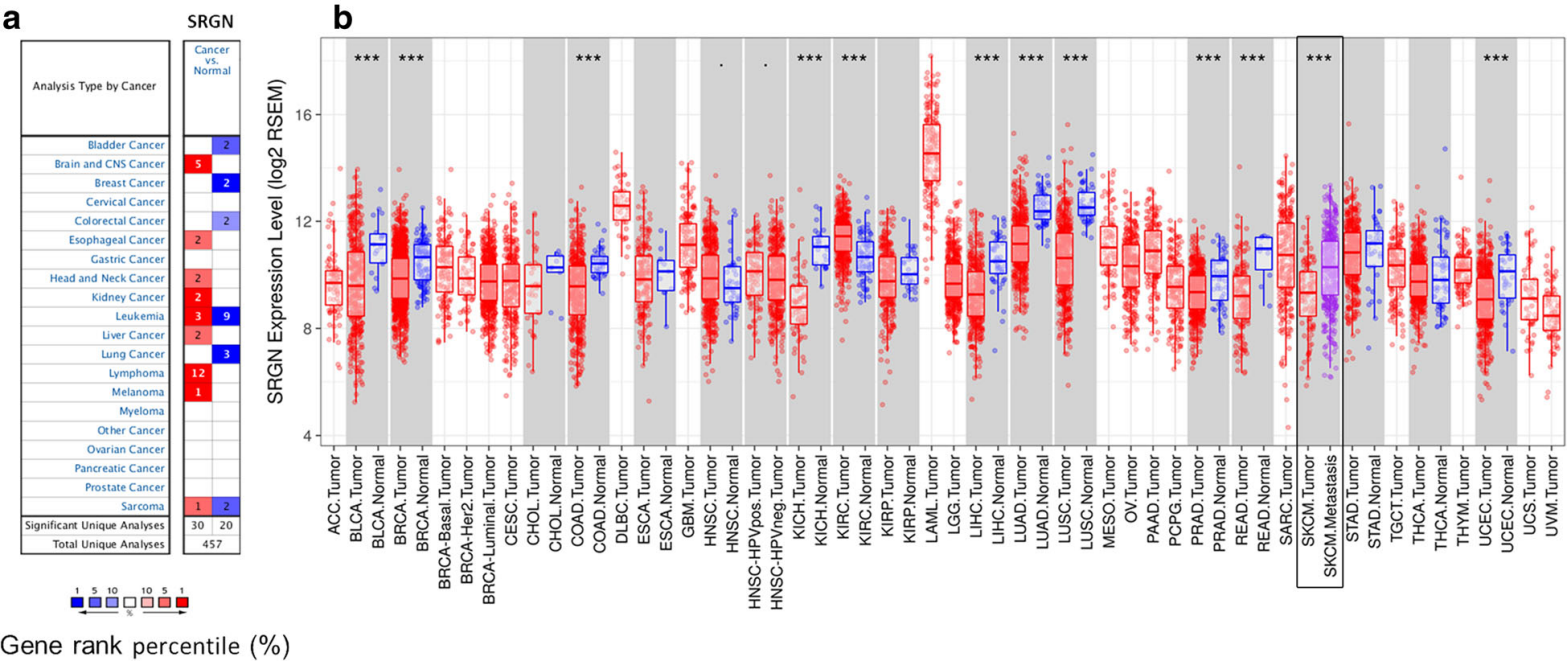
SRGN expression in different types of human cancers. **a** Different expression of SRGN between cancer and normal tissues in Oncomine database. **b** Different expression of SRGN between tumor and adjacent normal tissues across all TCGA tumors in TIMER database. SRGN, serglycin; ***P < 0.001

To explore the different expression of SRGN between tumor and normal tissues, we used Oncomine database for analysis. Comparing with normal tissues, higher expression of SRGN was found in brain and CNS (central nervous system) cancer, esophageal cancer, head and neck cancer, kidney cancer, liver cancer, lymphoma, and melanoma while lower expression was found in bladder cancer, breast cancer, colorectal cancer, and lung cancer (Fig. 1a). In addition, we also studied the different SRGN expression between tumor and adjacent normal tissues across all TCGA tumors. It was showed that SRGN was significantly highly expressed in KIRC (kidney renal clear cell carcinoma) and significantly lower expressed in BLCA (bladder urothelial carcinoma), BRCA (Breast invasive carcinoma), COAD (colon adenocarcinoma), KICH (kidney chromophobe), LIHC (liver hepatocellular carcinoma), LUAD (lung adenocarcinoma), LUSC (Lung squamous cell carcinoma), PRAD (prostate adenocarcinoma), READ (Rectum adenocarcinoma) and UCEC (Uterine Corpus Endometrial Carcinoma) (*P* < 0.001, Fig. 1b).

### Increased expression of SRGN in SKCM and SKCM-metastasis tissues

**Fig. 2:**
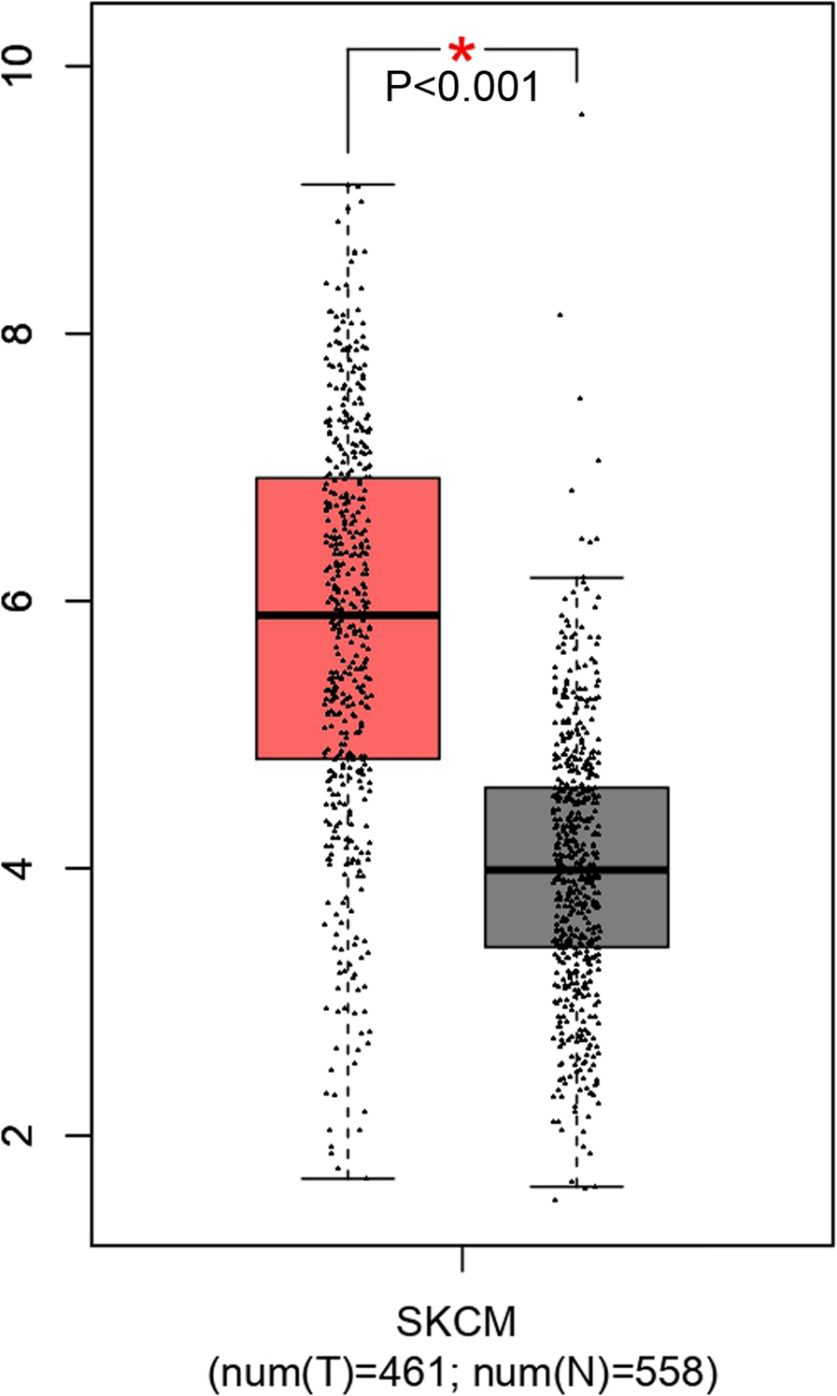
SRGN expression of normal and SKCM tissues in GEPIA database. SKCM, skin cutaneous melanoma; SRGN, serglycin;*P < 0.001

To better understand the role of SRGN in SKCM, we compared the expression of SRGN between SKCM (461samples) and normal (558samples) tissues in GEPIA database. It was shown that SRGN expression significantly higher in SKCM tissues than normal tissues (Fig. 2). Interestingly, we also found that the expression of SRGN was further increased in SKCM metastasis tissues (Fig. 1b) which indicates that SRGN may play a key role in the progression of SKCM.

### SRGN expression predicts the overall survival of SKCM and SKCM-metastasis patients

**Fig. 3:**
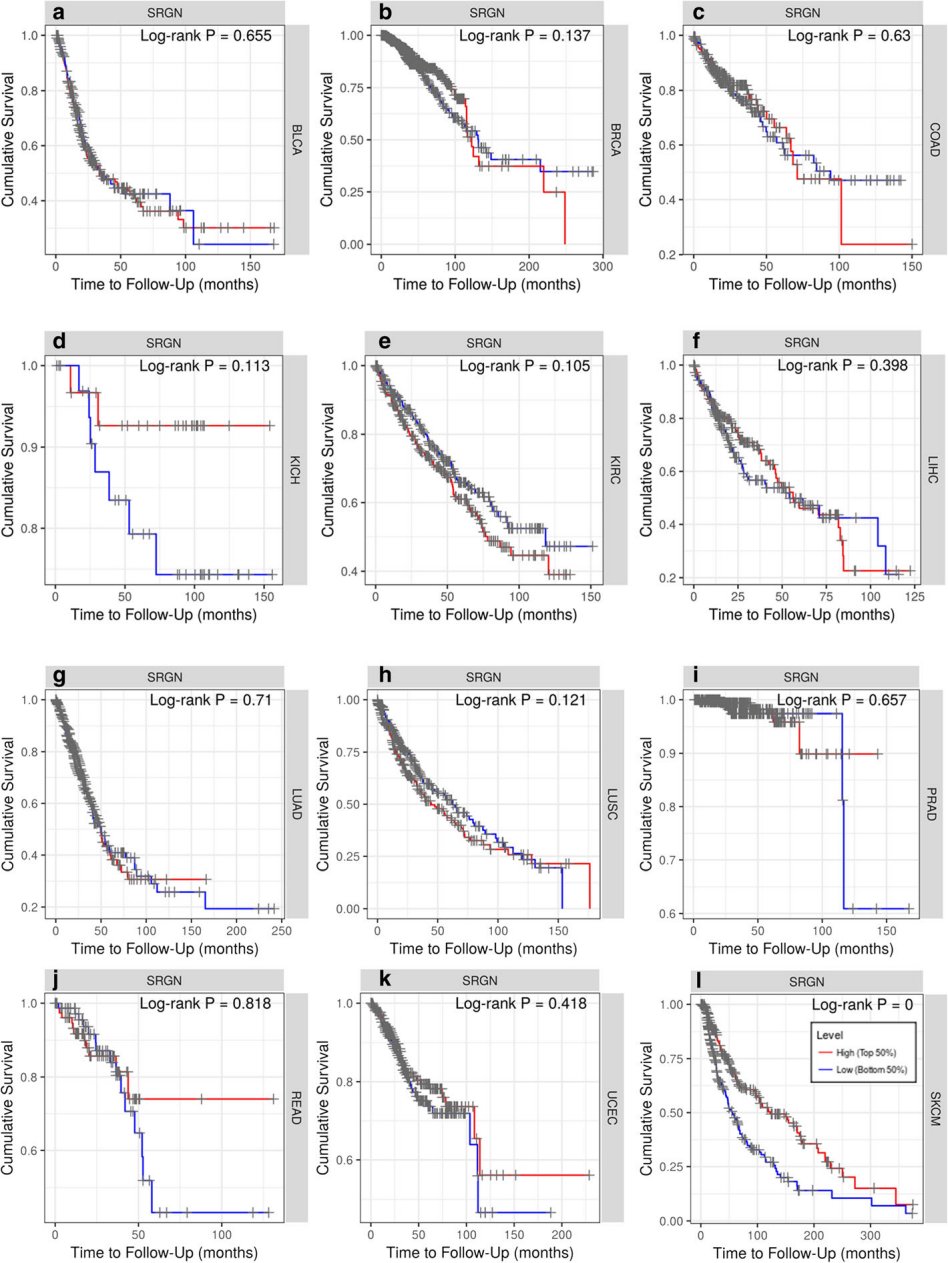
Kaplan-Meier survival curves comparing the high and low expression of SRGN in different types of cancer in TIMER database **(a-l)**. BLCA,bladder urothelial carcinoma; BRCA, Breast invasive carcinoma; COAD, colon adenocarcinoma; KICH, kidney chromophobe; KIRC, kidney renal clear cell carcinoma; LIHC, liver hepatocellular carcinoma; LUAD, lung adenocarcinoma; LUSC, Lung squamous cell carcinoma; PRAD, prostate adenocarcinoma; READ, rectum adenocarcinoma; UCEC,uterine corpus endometrial carcinoma; SKCM, skin cutaneous melanoma; SRGN, serglycin

**Fig. 4:**
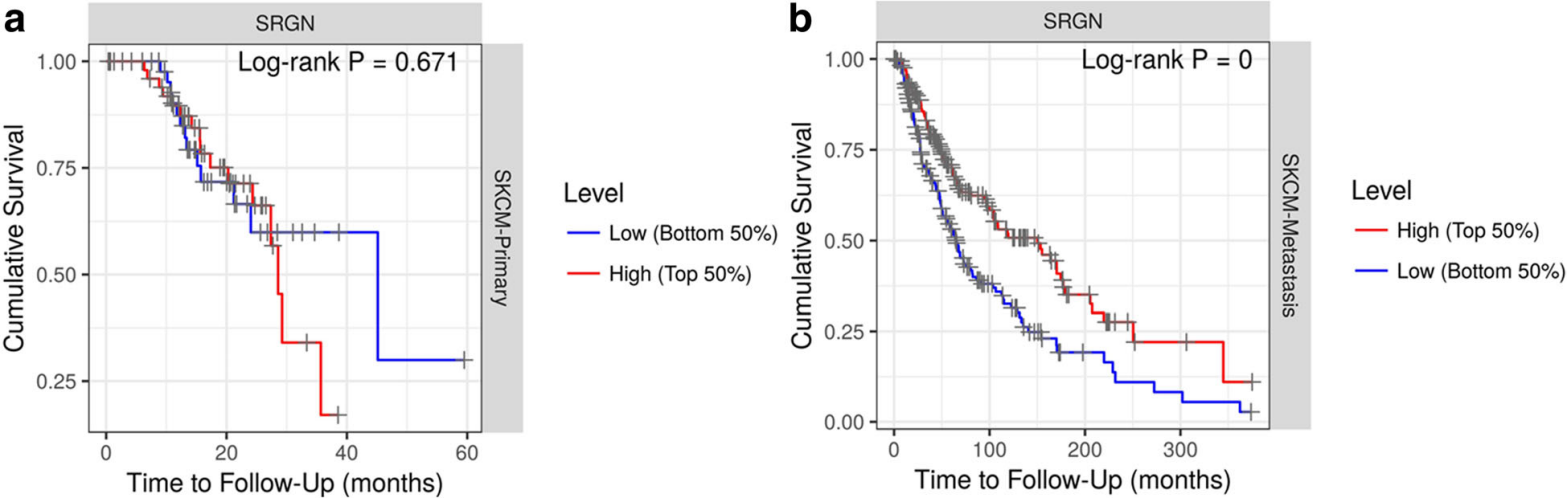
Kaplan-Meier survival curves comparing the high and low expression of SRGN in primary SKCM and metastatic SKCM. SKCM, skin cutaneous melanoma; SRGN, serglycin

We investigated the association between SRGN expression and survival of patients with different tumors. In TIMER database, after adjustment of age, gender, ethnicity, tumor stages and tumor purity, we found that lower SRGN expression was only significantly correlated with poor survival of SKCM patients (Fig. 3). In the database, the SKCM patients (393 cases, 187 dying) include primary (97 cases, 27 dying) and metastatic (296 cases, 160 dying) patients. Furthermore, we also explored the influence of SRGN expression on the survival of SKCM-primary (Fig. 4a) and SKCM-metastasis (Fig. 4b) patients. It was found that lower SRGN expression was only significantly associated with the survival rate of SKCM-metastasis patients not SKCM-primary patients (Fig. 4b). Therefore, it is conceivable that low SRGE expression is an independent risk factor for the survival of SKCM and SKCM-metastasis patients.

### SRGN expression is correlated with immune infiltrates in SKCM and SKCM-metastasis

**Fig. 5:**
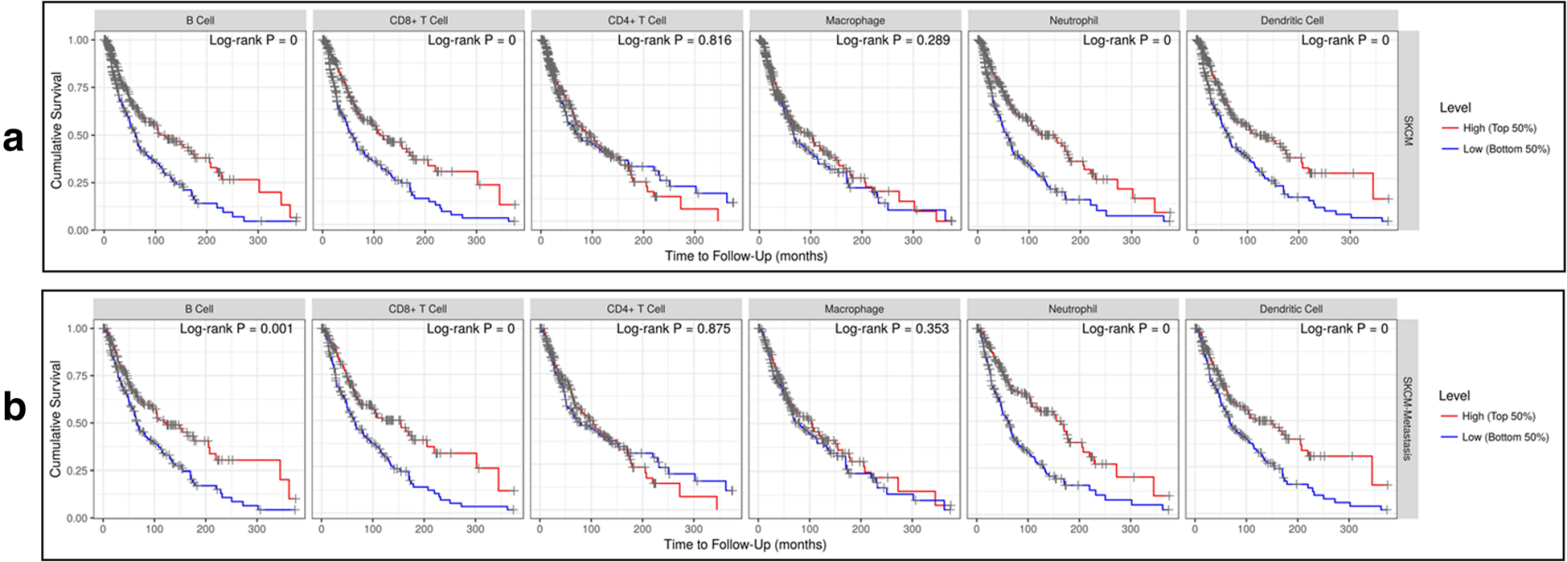
The relationship between immune infiltrates and survival rate of SKCM and SKCM-metastasis patients. Immune cells infiltrates was divided into high and low groups. SKCM, skin cutaneous melanoma

**Fig. 6:**
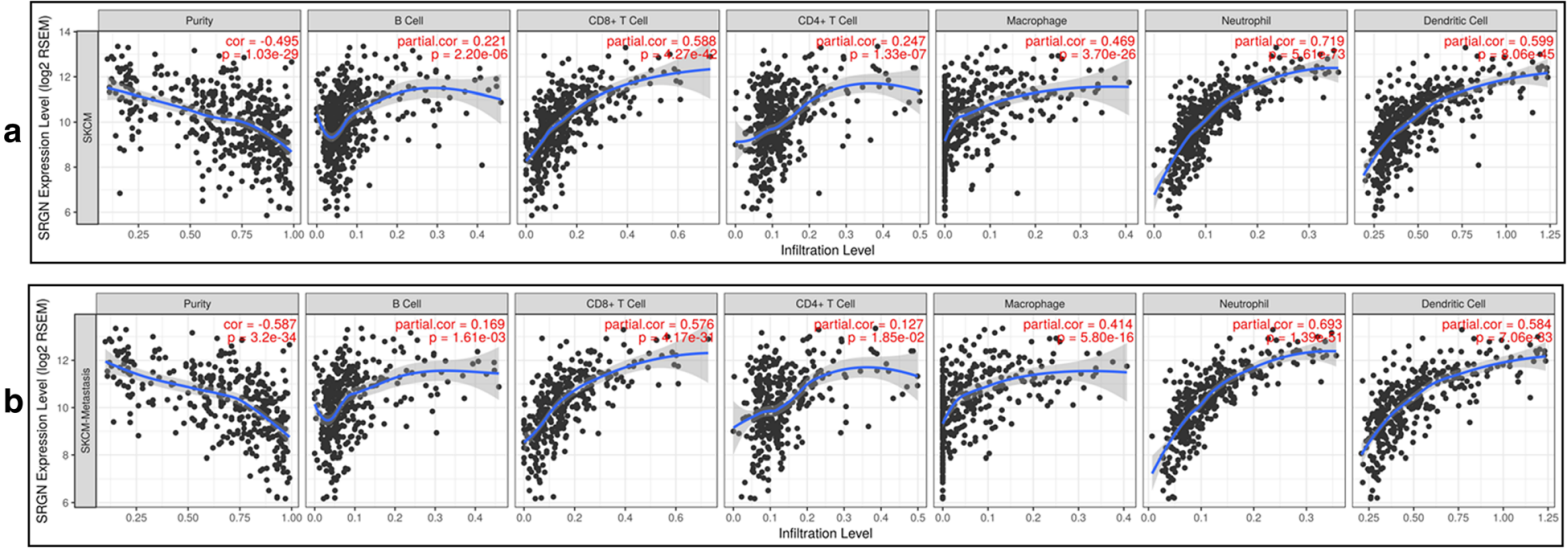
Correlation analyses of SRGN expression and immune infiltrates in SKCM and SKCM-metastasis patients. SKCM, skin cutaneous melanoma; SRGN, serglycin

It has been demonstrated that tumor-infiltrating lymphocytes grade is an independent predictor of survival and sentinel lymph node status in patients with melanoma [6]. In our study, we also analyzed the relationship between immune infiltrates and survival rate of SKCM and SKCM-metastasis patients. After the adjustment of clinical factors (age, gender, ethnicity, tumor stages and tumor purity), we found that the lower infiltrates of B cell, CD8^+^ T cell, Neutrophil, and Dendritic cell was significantly associated with a poor survival rate of SKCM and SKCM-metastasis patients (Fig. 5a-b) which is consistent with the previous study [6]. Then, we investigated whether SRGN expression was correlated with the immune infiltrates of SKCM and SKCM-metastasis. It was shown that SRGN expression had a significant positive correlation with the infiltrating levels of B cell, CD8^+^ T cell, CD4^+^ T cell, macrophage, neutrophil, and dendritic cell in SKCM (Fig. 6a) and SKCM-metastasis patients (Fig. 6b). Furthermore, we also explored the relationship between SRGN expression and immune marker genes of B cell, CD8^+^ T cell, Neutrophil, and Dendritic cell which we demonstrated having predict value for the survival of SKCM and SKCM-metastasis patients. It was found that most of the immune marker genes of B cell, CD8+ T cell, Neutrophil, and Dendritic cell were significantly associated with the SRGN expression (Table 1). The above findings imply that SRGN influences the survival of SKCM and SKCM-metastasis patients possibly through regulating the infiltration of immune cells.

**Table 1:** Correlation analysis between serglycin (SRGN) and gene markers of immune cells in skin cutaneous melanoma (SKCM) and SKCM-metastasis

Immune cell	Gene marker	SKCM		SKCM-metastasis	
		Cor	*P* value	Cor	*P* value
B cell	CD 19	0.364	< 0.001	0.248	< 0.001
	CD 79	0.331	< 0.001	0.248	< 0.001
CD8^+^ T cell	CD8A	0.538	< 0.001	0.510	< 0.001
	CD8B	0.499	< 0.001	0466	< 0.001
Neutrophils	CD66b (CEACAM8)	0.006	0.89	0.0110	0.833
	CD11b (ITGAM)	0.592	< 0.001	0.528	< 0.001
	CCR7	0.317	< 0.001	0.267	< 0.001
Dendritic cell	HLA-DPB1	0.515	< 0.001	0.521	< 0.001
	HLA-DQB1	0.458	< 0.001	0.445	< 0.001
	HLA-DRA	0.604	< 0.001	0.603	< 0.001
	HLA-DPA1	0.538	< 0.001	0.519	< 0.001
	BDCA-1(CD1C)	0.287	< 0.001	0.267	< 0.001
	BDCA-4 (NRP1)	0.408	< 0.001	0.326	< 0.001
	CD11c (ITGAX)	0.336	< 0.001	0.281	< 0.001

## Discussion

In the present study, we found that the expression of SRGN was positively associated with the immune infiltrates and the survival rate of SKCM and SCKM-metastasis patients. Our findings firstly highlight the important role of SRGN in SKCM and SCKM-metastasis. Furthermore, it implies that SRGN may be a new immune target for treating melanoma.

It is known that melanoma is a lethal skin cancer and highly metastatic. The metastasis is a major barrier for the treatment of melanoma. Recently, a great effort has been made for using immune therapies to treat SKCM [7, 26]. In our study we found that lower infiltrates of B cell, CD8^+^ T cell, Neutrophil, and Dendritic cell were associated with the worse survival of SKCM and SKCM-metastasis patients. Our findings are somewhat consistent with previous studies [5, 27, 28]. Using the mRNA sequencing data from TCGA, it was found that high expression of T-cell and B-cell signatures predicted improved overall survival of melanoma [27]. In latest study, Sara R. Selitsky et al. highlighted the important role of B cell in modulating the anti-tumor immune response in SKCM [5]. However, as for the Neutrophil and Dendritic cell infiltrates, there seems have different point of view against our findings [29, 30]. A former study once demonstrated that neutrophil infiltration and CD123+ dendritic cell infiltration in primary melanoma were independently associated with poor prognosis [29]. The difference may because in their study, they only used CD66b and CD123+ respectively for the marker of infiltrating neutrophils and dendritic cells which is not sufficient. In our study, we used different immune cell markers evaluation to explore the immune infiltrates in melanoma. It was found that most of the markers have a strong relationship with SRGN expression (Table 1). But it is known that these markers are only relatively specific for these immune cells, so SRGN may influence them differently. Another possible explanation is that we did not calculate the ratio of neutrophil to lymphocyte. Because it has been demonstrated that neutrophil to lymphocyte ratio is an independent predictor for the outcome of melanoma patients [31, 32]. Interestingly, in our study, we found that SRGN expression had a significant positive correlation with macrophage and CD4+T cells infiltration. But we didn’t find macrophage and CD4+T cells associated with a poor survival rate of SKCM and SKCM-metastasis patients. It may because SRGN influencing more function of macrophage and CD4+T cells than quantity or SRGN on macrophage and CD4+T cells infiltration might be coordinated by other signals to have less effect on the survival. Furthermore, in the present study, we can only evaluate survival rate based on different immune cell infiltrates and cannot analyze the conjoined effect of those immune cells. So, more studies are needed to explain the important role of immune infiltrates in melanoma.

SRGN is a proteoglycan that was first found in hematopoietic cells. Nowadays, it has been demonstrated that SRGN can be secreted by various cells including tumor cells [16]. In previous studies, most researches found that high SRGN expression was correlated with low survival rate of most tumors such as breast cancer [33], prostate cancer [34], colorectal cancer [15], nasopharyngeal carcinoma [17], glioma [35], and primary lung adenocarcinomas [36]. However, the role of SRGN in melanoma has not been explored before. In the present study, it was found that the expression of SRGN was increased in SKCM and SKCM-metastasis. Interestingly, we found that high SRGN expression was associated with improved survival which seems opposite to previous studies. It may because in SKCM, SRGN carries different glycosaminoglycan chains which depending on cell type can be either chondroitin sulfate, dermatan sulfate, heparan sulfate, and heparin [37]. Another possible explanation is that the increased SRGN lacks of the glycosaminoglycan attachment sites that failed to promote the cellular functions which has been found in breast cancer [33]. However, we infer that the most reasonable explanation is that SRGN regulates the immune infiltrates in SKCM and SKCM-metastasis. In our study, we found that the expression of SRGN was positively associated with the immune infiltrates which predicted improved overall survival of melanoma [27]. It has been demonstrated that serglycin-deficient mice were more susceptible to *T. spiralis* infection and displayed an unbalanced immune response compared to wild type mice [38]. Aging serglycin−/− mice spontaneously developed a massive enlargement of multiple lymphoid tissues, including spleen, Peyer’s patches, and bronchus-associated lymphoid tissue [39], suggesting that serglycin has functions in maintaining homeostasis of the body’s immune cell populations. Therefore, based on the previous studies, we inferred that regulating immune infiltrates is a key role of SRGN in SKCM and SKCM-metastasis. It is known that immune infiltrates can affect inflammation in cancer [40]. A series of evidence has been shown that serglycin is at the crossroad of inflammation and malignancy [41]. So another possible explanation is that serglycin may influence inflammation of melanoma through regulating immune infiltrates. However, all the results come from the cancer related database, more basic and large scale clinic studies are needed to verify our findings.

## Conclusions

In summary, SRGN expression is associated with the survival and immune infiltrates of SKCM and SKCM-metastasis patients. Targeting SRGN may be a new therapy for treating melanoma.

## Data Availability

Please contact the author Kun Zhang (zszhangk@hotmail.com) upon reasonable requests.

## Abbreviations

GEPIA: Gene Expression Profiling Interactive Analysis
SKCM: Skin cutaneous melanoma
SRGN: Serglycin
TCGA: The Cancer Genome Atlas
TIMER: Tumor IMmune Estimation Resource

## References

[1] Thompson JF, Scolyer RA, Kefford RF (2009). Cutaneous melanoma in the era of molecular profiling. Lancet..

[2] Fecher LA, Cummings SD, Keefe MJ, Alani RM (2007). Toward a molecular classification of melanoma. J Clin Oncol.

[3] Siegel R, Ward E, Brawley O, Jemal A (2011). Cancer statistics, 2011: the impact of eliminating socioeconomic and racial disparities on premature cancer deaths. CA Cancer J Clin.

[4] Bogunovic D, O'Neill DW, Belitskaya-Levy I, Vacic V, Yu YL, Adams S (2009). Immune profile and mitotic index of metastatic melanoma lesions enhance clinical staging in predicting patient survival. Proc Natl Acad Sci U S A.

[5] Selitsky SR, Mose LE, Smith CC, Chai S, Hoadley KA, Dittmer DP (2019). Prognostic value of B cells in cutaneous melanoma. Genome Med.

[6] Azimi F, Scolyer RA, Rumcheva P, Moncrieff M, Murali R, McCarthy SW (2012). Tumor-infiltrating lymphocyte grade is an independent predictor of sentinel lymph node status and survival in patients with cutaneous melanoma. J Clin Oncol.

[7] Hodi FS, O'Day SJ, McDermott DF, Weber RW, Sosman JA, Haanen JB (2010). Improved survival with ipilimumab in patients with metastatic melanoma. N Engl J Med.

[8] Topalian SL, Hodi FS, Brahmer JR, Gettinger SN, Smith DC, McDermott DF (2012). Safety, activity, and immune correlates of anti-PD-1 antibody in cancer. N Engl J Med.

[9] Toyama-Sorimachi N, Sorimachi H, Tobita Y, Kitamura F, Yagita H, Suzuki K (1995). A novel ligand for CD44 is serglycin, a hematopoietic cell lineage-specific proteoglycan. Possible involvement in lymphoid cell adherence and activation. J Biol Chem.

[10] Zernichow L, Abrink M, Hallgren J, Grujic M, Pejler G, Kolset SO (2006). Serglycin is the major secreted proteoglycan in macrophages and has a role in the regulation of macrophage tumor necrosis factor-alpha secretion in response to lipopolysaccharide. J Biol Chem.

[11] Kolset SO, Tveit H (2008). Serglycin--structure and biology. Cell Mol Life Sci.

[12] Niemann CU, Kjeldsen L, Ralfkiaer E, Jensen MK, Borregaard N (2007). Serglycin proteoglycan in hematologic malignancies: a marker of acute myeloid leukemia. Leukemia..

[13] Zhang Z, Deng Y, Zheng G, Jia X, Xiong Y, Luo K (2017). SRGN-TGFbeta2 regulatory loop confers invasion and metastasis in triple-negative breast cancer. Oncogenesis..

[14] Bouris P, Manou D, Sopaki-Valalaki A, Kolokotroni A, Moustakas A, Kapoor A (2018). Serglycin promotes breast cancer cell aggressiveness: induction of epithelial to mesenchymal transition, proteolytic activity and IL-8 signaling. Matrix Biol.

[15] Xu Y, Xu J, Yang Y, Zhu L, Li X, Zhao W (2018). SRGN promotes colorectal Cancer metastasis as a critical downstream target of HIF-1alpha. Cell Physiol Biochem.

[16] Chu Q, Huang H, Huang T, Cao L, Peng L, Shi S (2016). Extracellular serglycin upregulates the CD44 receptor in an autocrine manner to maintain self-renewal in nasopharyngeal carcinoma cells by reciprocally activating the MAPK/beta-catenin axis. Cell Death Dis.

[17] Li XJ, Ong CK, Cao Y, Xiang YQ, Shao JY, Ooi A (2011). Serglycin is a theranostic target in nasopharyngeal carcinoma that promotes metastasis. Cancer Res.

[18] Skliris A, Labropoulou VT, Papachristou DJ, Aletras A, Karamanos NK, Theocharis AD (2013). Cell-surface serglycin promotes adhesion of myeloma cells to collagen type I and affects the expression of matrix metalloproteinases. FEBS J.

[19] Theocharis AD, Karamanos NK (2019). Proteoglycans remodeling in cancer: Underlying molecular mechanisms. Matrix Biol.

[20] Rhodes DR, Kalyana-Sundaram S, Mahavisno V, Varambally R, Yu J, Briggs BB (2007). Oncomine 3.0: genes, pathways, and networks in a collection of 18,000 cancer gene expression profiles. Neoplasia..

[21] Li T, Fan J, Wang B, Traugh N, Chen Q, Liu JS (2017). TIMER: a web server for comprehensive analysis of tumor-infiltrating immune cells. Cancer Res.

[22] Tang Z, Li C, Kang B, Gao G, Li C, Zhang Z (2017). GEPIA: a web server for cancer and normal gene expression profiling and interactive analyses. Nucleic Acids Res.

[23] Li B, Li JZ (2014). A general framework for analyzing tumor subclonality using SNP array and DNA sequencing data. Genome Biol.

[24] Pan JH, Zhou H, Cooper L, Huang JL, Zhu SB, Zhao XX (2019). LAYN is a prognostic biomarker and correlated with immune infiltrates in gastric and Colon cancers. Front Immunol.

[25] Danaher P, Warren S, Dennis L, D'Amico L, White A, Disis ML (2017). Gene expression markers of tumor infiltrating leukocytes. J Immunother Cancer.

[26] Besser MJ, Shapira-Frommer R, Treves AJ, Zippel D, Itzhaki O, Hershkovitz L (2010). Clinical responses in a phase II study using adoptive transfer of short-term cultured tumor infiltration lymphocytes in metastatic melanoma patients. Clin Cancer Res.

[27] Iglesia MD, Parker JS, Hoadley KA, Serody JS, Perou CM, Vincent BG. Genomic Analysis of Immune Cell Infiltrates Across 11 Tumor Types. J Natl Cancer Inst. 2016;108(11). 10.1093/jnci/djw144 PMC5241901 27335052

[28] Gross S, Erdmann M, Haendle I, Voland S, Berger T, Schultz E, et al. Twelve-year survival and immune correlates in dendritic cell-vaccinated melanoma patients. JCI Insight. 2017;2(8). 10.1172/jci.insight.91438 PMC5396520 28422751

[29] Jensen TO, Schmidt H, Moller HJ, Donskov F, Hoyer M, Sjoegren P (2012). Intratumoral neutrophils and plasmacytoid dendritic cells indicate poor prognosis and are associated with pSTAT3 expression in AJCC stage I/II melanoma. Cancer..

[30] Nedelcu RI, Ion DA, Holeab CA, Cioplea MD, Brinzea A, Zurac SA (2015). Dendritic cells in melanoma - immunohistochemical study and research trends. Romanian J Morphol Embryol.

[31] Lino-Silva LS, Salcedo-Hernandez RA, Garcia-Perez L, Meneses-Garcia A, Zepeda-Najar C (2017). Basal neutrophil-to-lymphocyte ratio is associated with overall survival in melanoma. Melanoma Res.

[32] Kanatsios S, Melanoma Project M (2018). Li Wai Suen CSN, Cebon JS, Gyorki DE. Neutrophil to lymphocyte ratio is an independent predictor of outcome for patients undergoing definitive resection for stage IV melanoma. J Surg Oncol.

[33] Korpetinou A, Skandalis SS, Moustakas A, Happonen KE, Tveit H, Prydz K (2013). Serglycin is implicated in the promotion of aggressive phenotype of breast cancer cells. PLoS One.

[34] Korpetinou A, Papachristou DJ, Lampropoulou A, Bouris P, Labropoulou VT, Noulas A (2015). Increased expression of Serglycin in specific carcinomas and aggressive Cancer cell lines. Biomed Res Int.

[35] Roy A, Attarha S, Weishaupt H, Edqvist PH, Swartling FJ, Bergqvist M (2017). Serglycin as a potential biomarker for glioma: association of serglycin expression, extent of mast cell recruitment and glioblastoma progression. Oncotarget..

[36] Guo JY, Hsu HS, Tyan SW, Li FY, Shew JY, Lee WH (2017). Serglycin in tumor microenvironment promotes non-small cell lung cancer aggressiveness in a CD44-dependent manner. Oncogene..

[37] Skliris A, Happonen KE, Terpos E, Labropoulou V, Borset M, Heinegard D (2011). Serglycin inhibits the classical and lectin pathways of complement via its glycosaminoglycan chains: implications for multiple myeloma. Eur J Immunol.

[38] Roy A, Sawesi O, Pettersson U, Dagalv A, Kjellen L, Lunden A (2016). Serglycin proteoglycans limit enteropathy in Trichinella spiralis-infected mice. BMC Immunol.

[39] Wernersson S, Braga T, Sawesi O, Waern I, Nilsson KE, Pejler G (2009). Age-related enlargement of lymphoid tissue and altered leukocyte composition in serglycin-deficient mice. J Leukoc Biol.

[40] Grivennikov SI, Greten FR, Karin M (2010). Immunity, inflammation, and cancer. Cell..

[41] Korpetinou A, Skandalis SS, Labropoulou VT, Smirlaki G, Noulas A, Karamanos NK (2014). Serglycin: at the crossroad of inflammation and malignancy. Front Oncol.

